# Long-Term Clinical Remission on Benralizumab Treatment in Severe Eosinophilic Asthma: A Four-Year Real-Life Study

**DOI:** 10.3390/jcm14062075

**Published:** 2025-03-18

**Authors:** Carla Maria Irene Quarato, Pasquale Tondo, Donato Lacedonia, Piera Soccio, Dalila Pescatore, Maria Lisa Baccellieri, Giorgia Lepore, Maria Pia Foschino Barbaro, Giulia Scioscia

**Affiliations:** 1 Institute of Respiratory Diseases, Policlinico Riuniti of Foggia, 71122 Foggia, Italy; carlamariairene.quarato@gmail.com (C.M.I.Q.); pasquale.tondo@unifg.it (P.T.); donato.lacedonia@unifg.it (D.L.); giorgia.lepore@unifg.it (G.L.); giulia.scioscia@unifg.it (G.S.); 2 Department of Medical and Surgical Sciences, University of Foggia, 71122 Foggia, Italy; alilad.pes@gmail.com (D.P.); maria.baccellieri@unifg.it (M.L.B.); mariapia.foschino@unifg.it (M.P.F.B.)

**Keywords:** asthma, benralizumab, non-invasive biomarkers

## Abstract

**Background:** The current availability of monoclonal antibodies against key mediators of type-2 (T2) inflammation has led to a redefinition of the ultimate objectives of severe asthma treatment to a more composite concept of disease remission. **Objectives:** The aim of this real-life study was to estimate the percentage of patients who achieved clinical remission over 4 years of treatment with benralizumab, and to identify baseline predictors for the achievement of such a composite outcome in the long term. **Methods:** Data from a 4-year follow-up of 23 patients who were prescribed benralizumab as an add-on therapy because of uncontrolled severe eosinophilic asthma were retrospectively analyzed and compared. Clinical remission was considered to be “complete” if oral corticosteroid (OCS) use was not required, there were no exacerbations, an asthma control test (ACT) score ≥ 20 was achieved and a pre-bronchodilation percent predicted a forced expiratory volume in 1 s (FEV_1_%) ≥ 80%. Clinical remission was considered to be “partial” if OCS use was not required, plus at least two of the other three aforementioned criteria. **Results:** The overall percentage of patients who achieved clinical remission was 86.9% after 12 months, and 91.3% after 24 and 48 months of treatment. The rate of complete remission over partial remission increased over time. After 12 months of treatment, 65% of patients fulfilled the criteria for complete remission and 35.0% for partial remission. After 48 months of treatment, 71.4% of patients were in a status of complete remission and 28.6% in a status of partial remission. A long-term composite outcome of complete clinical remission was more likely to be achieved by severe eosinophilic asthma patients with comorbid nasal polyposis, bronchiectasis and osteoporosis, and with OCS dependency, a predicted pre-bronchodilation FEV_1_% ≥ 80% and a predicted FEF_25–75%_ < 65% at baseline. **Conclusions:** Our real-life experience suggests that treatment with benralizumab may allow the achievement and long-term maintenance of clinical remission in a high percentage of severe eosinophilic asthma patients, up to 4 years of follow-up.

## 1. Introduction

Asthma is a condition characterized by chronic airway inflammation, bronchial hyperreactivity and a variable limitation of expiratory airflow, leading to respiratory symptoms (i.e., wheezing, chest tightness and shortness of breath) which can vary in intensity over time [1]. Since variability in the expression of the disease is a fundamental feature of asthma, patients may become symptom-free either spontaneously or remain symptom-free with the appropriate inhalation therapy. However, patients with severe asthma have poor symptom control and suffer from recurrent asthma exacerbations, even if treated with high doses of inhaled corticosteroids (ICS) combined with long-acting beta-2-agonists (LABAs) and other controllers, such as long-acting muscarinic antagonists (LAMAs) and/or leukotriene receptor antagonists (LTRAs) [1,2]. This frequently forces severe asthmatics to resort to emergency department (ED) visits or hospitalization, and an intermittent or chronic use of oral corticosteroids (OCSs) in addition to their maximal maintenance therapy; this brings the risk of a whole series of long-term side effects, such as osteoporosis, cataracts, metabolic syndrome, type 2 diabetes, obesity, digestive disorders, insomnia and depression [2,3]. According to the International Study of Asthma and Allergies in Childhood (ISAAC) and the Global Asthma Network (GAN), asthma affects nearly 300 million people worldwide, with significant regional variations in prevalence and severity [4,5]. Although only an estimated 5–10% of asthmatics suffer from severe asthma, the disease implies very high healthcare costs due to medications, physician visits, hospitalizations and OCS side effects [6,7]. Nowadays, severe asthma is no longer considered as a single disease, but as a heterogeneous syndrome that includes different phenotypes whose main determinants appear to be allergy (allergic vs. non-allergic asthma), age of onset (“early-onset” vs. “late-onset” asthma) and different variably associated comorbidities, such as nasal polyposis, atopic dermatitis and obesity [1]. Chronic airway inflammation has been recognized as the major driver of asthma. Furthermore, the increasingly in-depth knowledge of the etiopathogenetic and molecular mechanisms of severe asthma has made it possible to identify at least two main inflammatory endotypes, namely “type-2 (T2)-high” and “type-2 (T2)-low” asthma. T2-high asthma is characterized by an increased production of interleukin IL-4, IL-5 and IL-13, which may be related to atopy and an allergic immune response or may be activated by “allergen-independent” signals involving innate lymphoid cells-2 (ILC-2) [8]. Several monoclonal drugs, collectively known as “biologics”, have been approved and are currently available for the treatment of such severe asthma endotype. By turning off key signals involved in type-2 inflammatory pattern, these biological agents proved to be particularly effective in preventing the onset of exacerbations, avoiding the use of oral corticosteroids and significantly improving the control of symptoms and the quality of life of patients affected by severe asthma [9]. These therapeutic possibilities are recently leading to a redefinition of the ultimate objectives of severe asthma management which would move from the pursuit of individual outcomes, such as symptom control and prevention of exacerbations, to a composite concept of remission of the disease. However, there is still no univocal, accepted and internationally shared definition of what “clinical remission” in asthma means. An international consensus of experts (Menzies-Gow et al. [8]) distinguished two types of remission by identifying “clinical remission” as a composite outcome, including the absence of significant asthma-related symptoms, lung function stabilization, patient/provider agreement regarding remission and no use of systemic corticosteroids, and “complete remission” as clinical remission plus the objective resolution of asthma-related inflammation and, if appropriate, bronchial hyperresponsiveness. Another expert consensus from the Severe Asthma Network in Italy (SANI) [9] gave two distinct clinical remission definitions by regarding it as “complete” if OCS use was not required for the treatment of asthma, there were no exacerbations, but there was reasonable symptom control and stable optimal lung function, or as “partial” if OCS use was not required for the treatment of asthma plus the achievement of at least two of the other three additional criteria. More recently, a consensus published by the American College of Allergy, Asthma and Immunology (ACAAI), the American Academy of Allergy, Asthma and Immunology (AAAAI) and the American Thoracic Society (ATS) [10] proposed a mandatory compliance with six criteria for defining clinical remission, including no severe exacerbations, no missed work or school days, stable and optimized pulmonary function, only low-to-medium doses of ICS use (or less), symptom control and no more than once a month of reliever therapy. It is important to add that the criteria included in all these definitions of clinical remission must be maintained for a long-term time frame of at least 12 months, unlike the previous concept of asthma control, which considered a maximum of 1 month. However, there is still no univocal, accepted and internationally shared definition of what “clinical remission” in asthma means. An international consensus of experts by Menzies-Gow et al. [11] distinguished two types of remission by identifying “clinical remission” as a composite outcome including absence of significant asthma-related symptoms, lung function stabilization, patient/provider agreement regarding remission, and no use of systemic corticosteroids, and “complete remission” as clinical remission plus objective resolution of asthma-related inflammation and, if appropriate, bronchial hyperresponsiveness. Another expert consensus from the Severe Asthma Network in Italy (SANI) [12] gave two distinct clinical remission definitions by regarding it as “complete” in case of no-need for OCS for the treatment of asthma, lack of exacerbations, symptom control and a stable optimal lung function or as “partial” in case of no-need for OCS for the treatment of asthma plus the achievement of at least two of the other three additional criteria. More recently, a consensus published by the American College of Allergy, Asthma and Immunology (ACAAI), the American Academy of Allergy, Asthma and Immunology (AAAAI) and the American Thoracic Society (ATS) [13] proposed the mandatory compliance with six criteria for defining clinical remission, including no severe exacerbations, no missed work or school days, stable and optimized pulmonary function, only low-medium doses ICS use (or less), symptom control and need for reliever therapy no more than once a month. It is important to add that the criteria included in all these definitions of clinical remission must be maintained for a long-term time frame of at least 12 months, unlike the previous concept of asthma control which considered a maximum of 1 month.

Benralizumab is a humanized afucosylated monoclonal antibody (IgG1, kappa) that binds with high affinity and specificity to the alpha subunit of the human IL-5 receptor (IL-5Rα), expressed specifically on the surface of eosinophils and basophils. This prevents the interaction between IL-5 and its receptor, so that eosinophil activation, maturation, recruitment and survival are inhibited. The absence of fucose in the Fc domain of benralizumab also determines high affinity for FcɣRIII receptors on immune effector cells, including natural killer (NK) cells. This effect causes apoptosis of eosinophils and basophils via enhanced antibody-dependent cell-mediated cytotoxicity (ADCC), which reduces eosinophilic inflammation [10,14,15]. In the two pivotal phase III randomized clinical trials (RCTs), SIROCCO [16] and CALIMA [17], benralizumab demonstrated to reduce the frequency of exacerbations and improve lung function in patients with inadequately controlled severe eosinophilic asthma (SEA). In the ZONDA [18] and PONENTE [19] trials, benralizumab also demonstrated a significant effect in reducing both the use of OCS and the exacerbation rate. In the real-life long-term ANANKE study [20], benralizumab demonstrated to be effective in decreasing exacerbations, eliminating OCS dependency and improving lung function and asthma control after 96 weeks of treatment. The aim of our real-life study was to estimate the proportion of patients who achieve a partial or complete clinical remission (according to the definition given by the SANI expert consensus) after 12, 24, 36 and 48 months of treatment and to identify baseline predictors of long-term complete remission.

## 2. Materials and Methods

This was a retrospective analysis of real-life data pertaining to 23 asthma patients who were prescribed benralizumab in our accredited severe asthma center of the University Polyclinic of Foggia as an add-on therapy because of uncontrolled SEA. The follow-up period covered the first 4 years of treatment.

Inclusion criteria were the following: age ≥ 18 years, lack of asthma control despite maximal inhaled treatment (according to STEP 4–5 of GINA guidelines [1]) or OCS therapy for at least 6 months during the previous year and a blood eosinophil count ≥ 150 cells/mcL at enrolment or ≥300 cells/mcL in the previous year. Exclusion criteria were a lack of adherence to the current asthma maintenance therapy and/or an incorrect inhaler technique. All enrolled patients were biologic naïve.

Benralizumab was prescribed at the licensed dose of 30 mg, administered as a subcutaneous injection once every 4 weeks for the first 3 doses and then every 8 weeks thereafter. Patients were visited at baseline (T_0_) and after 12 (T_12_), 24 (T_24_), 36 (T_36_) and 48 (T_48_) months of treatment. Baseline was set at the time of benralizumab prescription. At each visit the following parameters were recorded: need for OCSs, required dose of oral prednisone, number of exacerbations during the previous year, asthma control test (ACT) score, pre-bronchodilator forced expiratory volume in the 1st second (FEV_1_), forced mid-expiratory flow between 25% and 75% of FVC (FEF_25−75%_), fractional exhaled nitric oxide at a flow rate 50 mL/s (FeNO_50_), blood eosinophil count and total IgE levels. All 23 enrolled patients completed the entire 48-month period, without any dropping out from the study.

The study followed the principles of the Declaration of Helsinki and was approved by the local ethics committee of the University Polyclinic of Foggia (institutional review board approval N°17/CE/12 June 2014).

### 2.1. Asthma Control Test (ACT)

The asthma control test (ACT) is a 5-item survey scoring the frequency of general asthma symptoms, the use of rescue medications and the effect of asthma on daily functioning, with an overall self-assessment of asthma control. An ACT score > 20 indicated well-controlled asthma.

### 2.2. Pulmonary Function Tests

Spirometry was performed according to 2005 ERS/ATS standard guidelines by a calibrated spirometer (Sensormedics, Milan, Italy). Pre-bronchodilator spirometry was conducted at baseline, T_12_, T_24_, T_36_ and T_48_ to measure FEV_1_, FVC and FEF_25−75%_. Values were expressed in liters (L) and as a percentage of predicted according to the equations of Quanjer and Stocks. Post-bronchodilator spirometry was conducted at baseline to determine the reversibility of airflow limitation and confirm the diagnosis of asthma. Small airway flow limitation was judged as FEF 25–75% values less than 65% of predicted [21].

### 2.3. Inflammatory Markers

Blood tests for each patient were performed and examined during each visit to record the circulating eosinophil count and total IgE levels. In addition, a FeNO_50_ measurement was performed using a FENO+ device (Medisoft, Padova, Italy). A blood eosinophil count > 300 cell/mcL and a FeNO_50_ level > 50 parts per billion (ppb) were regarded as markers of high T2 inflammation [1,22].

### 2.4. Definition of Clinical Remission

For greater simplicity in routine clinical use, we have chosen to use the definition of clinical remission given by the SANI expert consensus [12]. Clinical remission was considered as “complete” in case of no need of OCS use to maintain control, an absence of exacerbations, ACT score ≥ 20 and pre-bronchodilation FEV_1_ ≥ 80% of the predicted value, and as “partial” in case of no need of OCS use, plus at least two of the other three aforementioned criteria.

### 2.5. Statistical Analysis

Numerical data are expressed as mean ± standard deviation (SD) and categorical data as the number (*n*) and percentage (%) of patients. The Kolmogorov–Smirnov and Shapiro–Wilk tests were used to assess data distribution. Paired student’s *t* test was used to compare countable variables, while the Fisher exact test was used for dichotomous variables. The odds ratio between baseline characteristics of the patients and the achievement of complete remission compared to partial remission at T_48_ was calculated with a confidence interval of 95%. A *p* < 0.05 was considered statistically significant. All data were analyzed using GraphPad (version 8, GraphPad Software Inc., San Diego, CA, USA).

## 3. Results

### 3.1. Patients’ Characteristics at Baseline

Baseline characteristics of the 23 SEA patients included in the study are detailed in Table 1.

The mean age at T_0_ was 58.30 ± 10.26 years and there were 16 females (69.6%) and 7 males (30.4%). The mean BMI was 27.25 ± 5.00 kg/m^2^. Prevalence of smoking habit (ex or current) was 21.7%. Self-reported mean age of asthma symptom onset was 34.26 ± 12.95 years, with childhood onset in 6 cases (26.1%) and adult onset in 17 cases (73.9%). An atopic state was documented via positive prick tests in 17 patients (73.9%).

Asthma-associated comorbidities were nasal polyposis in 14 cases (60.9%), osteoporosis in 12 cases (52.2%), gastroesophageal reflux in 10 cases (43.5%), bronchiectasis in 8 cases (34.9%), ASA sensitivity in 3 cases (13.0%), anxiety and depression in 3 cases (13.0%), OSAS in 2 cases (8.7%) and atopic dermatitis in 2 cases (8.7%).

Baseline asthma maintenance treatment included high dose ICS/LABA plus a LAMA in 15 cases (65.2%), high dose ICS/LABA plus a LAMA and an oral LTRA in 6 cases (26.1%) and high dose ICS/LABA plus a LTRA in 2 cases (8.7%). In addition, 21 patients (91.3%) experienced at least one asthma exacerbation in the previous year, with a mean of 2.61 ± 1.33 exacerbations/year (range: 0–8 exacerbations/year); also, 13 patients (56.5%) were OCS-dependent, and the mean daily intake of oral prednisone was 18.48 ± 12.85 mg. The mean ACT score was 14.57 ± 2.45.

The mean pre-bronchodilation FEV_1_ at T_0_ was 1.95 ± 0.62 L, corresponding to a mean FEV_1_% of the predicted value of 66.52 ± 15.94. The mean blood eosinophil count was 670.87 ± 237.26 cells/mcL, with 22 patients (95.6%) showing a peripheral eosinophil value > 300 cell/mcL. The mean FeNO_50_ was 48.43 ± 14.79 ppb, with 9 patients (39.1%) showing a value > 50 ppb. The mean total IgE count was 235.18 ± 219.65 kU/L.

### 3.2. Effect of Benralizumab on Individual Outcomes That Comprise the Definition of Clinical Remission

The overall effect of benralizumab on individual components of clinical remission is shown in Table 2.

#### 3.2.1. Effect of Benralizumab in Reducing Exacerbation

Two patients reported no exacerbations at baseline but were prescribed the biologic because they had been forced to take OCSs for at least 6 months in the previous year.

**Figure 1:**
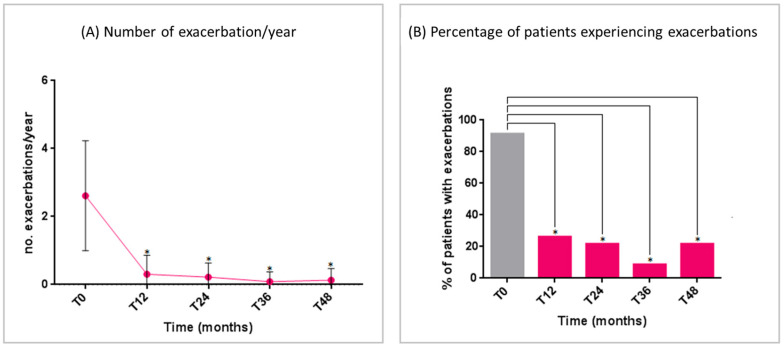
Benralizumab effectiveness on: (**A**) number of exacerbations/year (mean with range); (**B**) percentage of patients experiencing exacerbations. Statistically significant differences vs. baseline are highlighted with *. Abbreviations: T_0_ = baseline; T_12_ = 12 months; T_24_ = 24 months; T_36_ = 36 months; T_48_ = 48 months.

The mean number of exacerbations significantly decreased from T_0_ to T_12_ and remained low over the entire study period (Figure 1A). The number of patients experiencing asthma exacerbations significantly reduced from T_0_ to all time points (Figure 1B).

#### 3.2.2. Effect of Benralizumab in Reducing OCS Dependence

**Figure 2:**
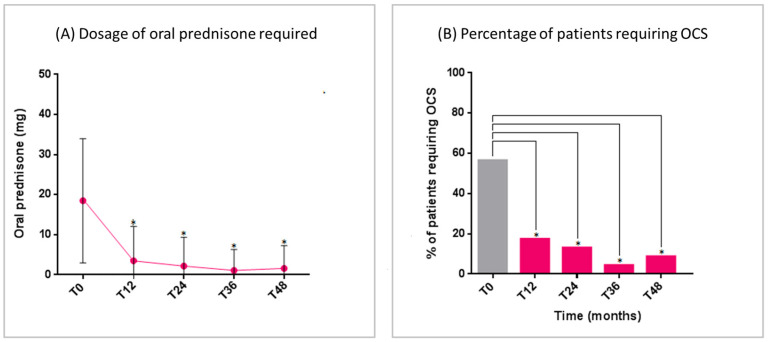
Benralizumab effectiveness on: (**A**) Dosage of oral prednisone required (mean with SD); (**B**) Percentage of patients requiring OCS. Statistically significant differences vs. baseline are highlighted with *. Abbreviations: T_0_ = baseline; T_12_ = 12 months; T_24_ = 24 months; T_36_ = 36 months; T_48_ = 48 months; OCS = oral corticosteroids.

The mean dose of daily oral prednisone intake significantly reduced over the study period, with a concomitant reduction in the number of OCS-dependent patients (Figure 2A,B). At T_48_, only two patients required a short burst of oral prednisone for asthma exacerbations.

#### 3.2.3. Effect of Benralizumab in Improving Respiratory Function

**Figure 3:**
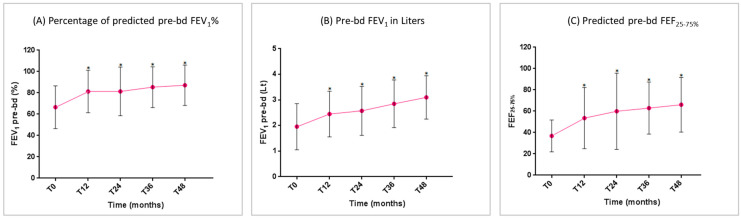
Benralizumab effectiveness on: (**A**) percentage of predicted pre-bd FEV_1_% (mean with SD); (**B**) pre-bd FEV_1_ in liters (mean with SD); (**C**) predicted pre-bd FEF_25–75%_ (mean with SD). Statistically significant differences vs. baseline are highlighted with *. Abbreviations: T_0_ = baseline; T_12_ = 12 months; T_24_ = 24 months; T_36_ = 36 months; T_48_ = 48 months; FEV_1_ = forced expiratory volume in 1 s; FEF_25–75%_ = forced expiratory flow between 25% and 75% of vital capacity.

The predicted pre-bronchodilation FEV_1_% began to improve in a statistically significant manner at T_12_. This improvement maintained statistical significance compared to the baseline over all of the study period (Figure 3A). The mean FEV_1_ gain vs. T_0_ was 1145 ± 258 mL at T_48_ (*p* < 0.0001) (Figure 3B). A significant improvement was also recorded regarding the small airway flow, with a progressive increase in the mean FEF_25–75%_ (Figure 3C).

#### 3.2.4. Effect of Benralizumab in the Asthma Control Test (ACT) Score

**Figure 4:**
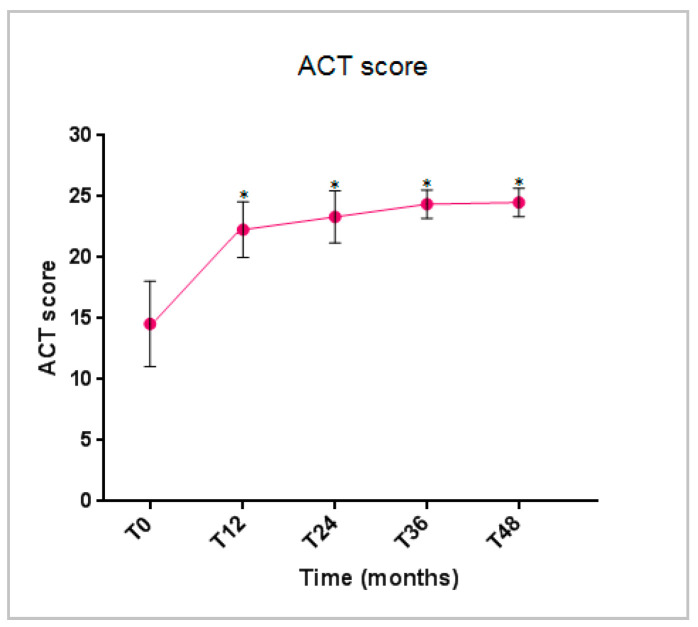
Benralizumab effectiveness on the ACT score (mean with SD). Statistically significant differences vs. baseline are highlighted with *. Abbreviations: T_0_ = baseline; T_12_ = 12 months; T_24_ = 24 months; T_36_ = 36 months; T_48_ = 48 months; ACT = asthma control test score.

The ACT score started to demonstrate a statistically significant improvement at 12 months and remained either stable or further increased throughout the treatment period, indicating a sustainable and good control of asthma (Figure 4).

### 3.3. Effects of Benralizumab on Markers of Asthma-Related T2 Inflammation

The overall effect of benralizumab on markers of asthma-related T2 inflammation is resumed in Table 3.

**Figure 5:**
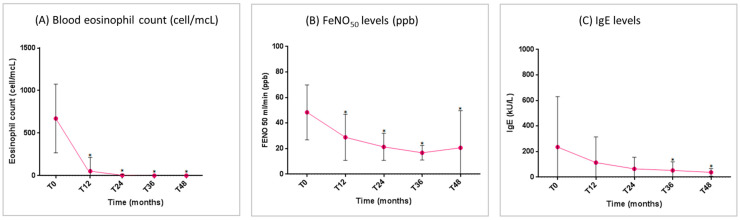
Benralizumab effectiveness on markers of asthma-related T2 inflammation: (**A**) blood eosinophil count (mean with SD); (**B**) FeNO_50_ levels (mean with SD); (**C**) total IgE levels (mean with SD). Statistically significant differences vs. baseline are highlighted with *. Abbreviations: T_0_ = baseline; T_6_ = 6 months; T_12_ = 12 months; T_24_ = 24 months; T_36_ = 36 months; T_48_ = 48 months; FeNO_50_ = fractional exhaled nitric oxide at a flow rate of 50 mL/s; IgE = immunoglobulin E.

The mean blood eosinophil count and FeNO_50_ levels showed a significant reduction from T_0_ to T_48_ (Figure 5A,B). Total IgE levels decreased gradually throughout the study period. The reduction was non-significant in the first two years, but became statistically significant starting from the third year of treatment (Figure 5C).

### 3.4. Clinical Remission Rates After Biological Therapy with Benralizumab

**Figure 6:**
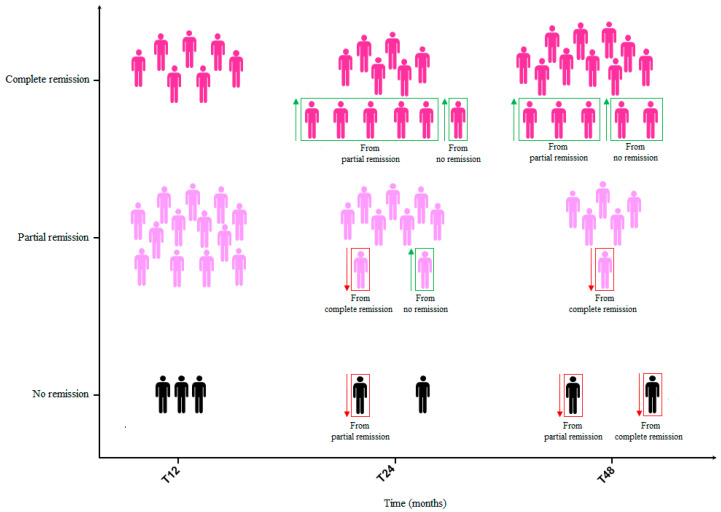
Patients on complete and partial remission with benralizumab at T_12_, T_24_ and T_48_. Abbreviations: T_12_ = 12 months; T_24_ = 24 months; T_36_ = 36 months; T_48_ = 48 months.

A total of twenty patients (86.9%) achieved remission after 1 year of benralizumab therapy. Among them, seven (35.0%) fulfilled the criteria for complete remission and thirteen (65.0%) had partial remission. After 2 years of treatment, a total of twenty-one patients (91.3%) were in a status of clinical remission. Among twelve patients (57.1%) who showed complete remission at T_24_, six maintained this condition from T_12_, five came from a partial remission status at T_12_ and one achieved it de novo. Among nine patients (42.8%) who showed partial remission at T_24_, seven maintained this condition from T_12_, one came from a complete remission status at T_12_ and one achieved it de novo. One patient lost their remission status from the partial remission group due to an exacerbation that required a short burst of OCS. A total of twenty-one patients (91.3%) showed clinical remission after 4 years of treatment. Among fifteen patients (71.4%) with complete remission at T_48_, ten maintained this condition from T_24_, three came from a partial remission status at T_12_ and two achieved it de novo. Among six patients (28.6%) with partial remission at T_48_, five maintained this condition from T_24_ and one came from a complete remission status at T_24_ due to an infectious exacerbation requiring antibiotic treatment. One patient lost their remission status from the partial remission group, and another patient lost their remission status from the complete remission group due to asthma exacerbations that required OCS use (Figure 6).

### 3.5. Baseline Predictors of SEA Complete Clinical Remission After 48 Months of Treatment with Benralizumab

Baseline predictors for achievement/maintenance of complete remission compared to partial remission after 4 years of treatment with benralizumab are analyzed in Table 4.

Two female patients were not included in the analysis because they lost the remission status at T_48_ from a partial and complete remission at T_24_, respectively. The cause of the loss of remission in both cases was the occurrence of asthma exacerbations that required OCS use. The patient who lost the state of remission at T_48_ from that of partial remission at T_24_ was a former smoker with atopy, and was burdened by OCS dependency already at the baseline. The patient who lost the state of remission at T_48_ from that of complete remission at T_24_ had comorbid atopy and nasal polyposis.

**Figure 7:**
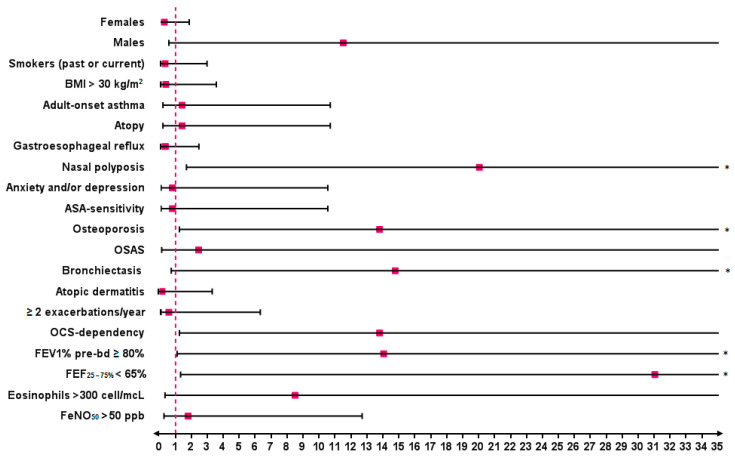
Strength of association in terms of odds ratio (OR) between baseline characteristics and clinical remission (complete and partial) vs. no remission after 4 years of treatment with benralizumab. Statistically significant results are highlighted with *. Abbreviations: BMI = body mass index; ASA sensitivity = sensitivity to aspirin; OSAS = obstructive sleep apnea syndrome; FEV_1_ = forced expiratory volume in 1 s; FEF_25–75%_ = forced expiratory flow between 25% and 75% of vital capacity; FeNO_50_ = fractional exhaled nitric oxide at a flow rate of 50 mL/s.

Predictor factors for the maintenance or achievement of complete remission, compared to partial remission at T_48_, were comorbid nasal polyposis (OR 20.00, 95% CI: 1.65–241.90; *p* = 0.01), osteoporosis (OR 13.75, 95% CI: 1.21–156.70; *p* = 0.04) and bronchiectasis (OR 14.73, 95% CI: 0.70–308.10; *p* = 0.04). Other factors positively associated with completing remission at T_48_ were OCS dependency (OR 13.75, 95% CI: 1.21–156.70; *p* = 0.04), a predicted pre-bronchodilation FEV_1_% ≥ 80% (OR 14.00, 95% CI: 1.06–185.60; *p* = 0.01) and a predicted FEF_25–75%_ < 65% (OR 31.00, 95% CI: 1.28–747.60; *p* = 0.01) at baseline (Figure 7).

## 4. Discussion

According to results of our study, the add-on treatment with benralizumab in uncontrolled SEA appeared effective in allowing the achievement of the outcomes included in the definition of clinical remission given by the SANI expert consensus [12], as well as in maintaining the obtained benefits in a follow-up period of 48 months. Clinical remission was evaluated in previous studies, both RCT and in real life. However, the criteria defining the remission status used in the various studies published so far have been somewhat arbitrary and not unequivocally agreed upon. According to results of a post-hoc analysis [23], almost 14.8% of patients enrolled in the SIROCO and CALIMA trials and 22.5% of those in the ZONDA study achieved early clinical remission (defined as a composite outcome of no exacerbations and OCS use, asthma symptom control and a pre-bronchodilator FEV_1_ increase ≥ 100 mL) at 6 months of treatment with benralizumab. An integrated analysis of the 1-year data from SIROCCO and CALIMA trials with data from the Phase III BORA safety extension study indicated that the efficacy improvements observed in the first year of treatment with benralizumab were maintained over a 2-years follow-up [24]. According to data of the international (Canada, Italy, Spain and UK) real-life XALOC-1 study [25], 43% out of 797 SEA patients showed zero exacerbations, had no need for maintenance OCS use, and achieved asthma symptom control after 12 months of benralizumab use. In the Spanish real-world ORBE II study [26], almost 43.7% of a total of 204 SEA patients on 1-year treatment with benralizumab reached a four criteria definition of clinical remission consisting in no exacerbations, no maintenance OCS use, an ACT score ≥ 20 and a pre-bronchodilator FEV_1_ increase ≥ 100 mL. Using a four criteria definition comprising absence of exacerbations, complete interruption of OCS therapy, ACT score of at least 20 and pre-bronchodilator FEV1 of at least 80% predicted, Pelaia et al. [27] documented a sustained remission rate of 42.1% in a cohort of 164 SEA patients after 2 years of treatment with benralizumab. Pini et al. [28] recently published a longer real-life study including up to 36 months of follow-up. Similar to our study, these authors used the criteria detailed by Canonica et al. [12] to establish the number and percentages of patients reaching either partial or complete remission, assessing an overall rate of 84.31% for long-term remission in a cohort of 108 SEA patients. Starting from this background, we further investigated the efficacy of benralizumab in inducing a state of clinical remission in a population of 23 SEA patients over a follow-up period of 48 months, observing that the rate of patients achieving such outcome increased over time. Indeed, the overall percentage of patients who achieved a remission status was 86.9% after 12 months of treatment and the rate of clinical remission further increased to 91.3% at both 24 and 48 months. Furthermore, the rate of complete remission over partial remission also increased. Among a total of 20 patients (86.9%) who achieved remission at T_12_, 65.0% fulfilled criteria for complete remission and 35.0% for partial remission. 21 patients (91.3%) reached clinical remission at T 48, of which 71.4% were in a status of complete remission and 28.6% in a status of partial remission. Interestingly, all patients achieved remission at least once during the study period. One patient went out of partial remission at T_24_ but gained complete remission at T_48_ and two patients lost the status of remission (partial and complete, respectively) at T_48_. The cause of the loss of remission in both cases were asthma exacerbations that required OCS use. This highlights that clinical remission is a rather dynamic condition and underlines the importance of long-term monitoring of asthma patients undergoing biological treatment. Anyway, asthma’s evolving nature, with potential changes in its specific driving cellular and molecular processes, may alter the patient’s inflammatory profile and attenuate the effectiveness of biologic therapies in the long-term, thus worsening the clinical response [29,30]. This implies the need of a running reassessment of the biomarker profile and therapeutic approach [30]. With regard to individual components defining remission, benralizumab made it possible to induce a statistically significant reduction of asthma exacerbations that persisted through the 48-months study period (the mean number of asthma exacerbations significantly decreased at T_12_ and remained low at all time points) and to reduce oral prednisone to the point of discontinuing it in more than a third of OCS-dependent patients (4 out of 13, 30.8%) after 12 months of treatment. The predicted pre-bronchodilator FEV_1_% significantly increased at T_12_, with a further increase although not statistically significant—over the 4-years follow-up and a remarkable mean +1145 ± 258 mL gain at T_48_. A positive and significant effect over time was observed also on small airways flow limitation. Interestingly, this data is in line with what has been documented by other authors [31,32]. At the same time, we observed a statistically significant improvement in asthma control in terms of ACT score. Considering the effect of benralizumab on markers of asthma-related type-2 inflammation, a remarkable reduction in blood eosinophil count was obtained after 12 months of treatment and maintained on long-term. This was an expected result taking into account the mechanism of action of this biologic drug [10,14,15]. Furthemore, a marked and sustained reduction in blood eosinophils count was assessed in numerous other studies [27,28,32]. Likewise, also FeNO, another airway type-2 inflammation marker, significantly decreased in the short time and remained low up to 48 months. Finally, the gradual decrease in total IgE levels, which reached statistical significance starting from the third year of treatment, could be related to a long-term modulation in the number of blood basophils induced by NK cells via enhanced antibody-dependent cell-mediated cytotoxicity [33], but studies confirming this hypothesis are needed. A predictor factor for the maintenance or achievement of complete remission compared to partial remission at T_48_, was comorbid nasal polyposis.

Other real-world studies reported that benralizumab brought greater benefits in SEA patients with comorbid CRSwNP [27,28,34]. A possible explanation is that both asthma and chronic rhinosinusitis with nasal polyps (CRSwNP) share the same underlying pathophysiological mechanisms, with type-2 inflammation being the cornerstone of both airway disorders [35]. As eosinophils are recognized as one of the main type-2 effectors, the remarkable reduction in blood eosinophil count obtained under treatment in our SEA patients indirectly reflects an important effect of benralizumab on turning off type-2 inflammatory cascade [36]. As a further confirm, such reduction in blood eosinophils was accompanied by a persistent reduction in FeNO. In this regard, Santomasi et al. [37] recorded also a significant reduction in the percentage of nasal eosinophils on nasal cytology and in the nasal polyps score (NPS) by nasal endoscopy in a cohort of 17 SEA patients with CRSwNP after one year of biological therapy with benralizumab. It would be very interesting to explore the achievement and maintenance of these results in a longer follow-up with further studies. Patients with bronchiectasis, together with patients with OCS-dependence and patients with reduced FEF_25–75%_ at T_0_, also showed a greater propensity to achieve complete remission at T_48_.

On the contrary, results of a real-world multicentre study by Campisi et al. [38] suggested a negative impact of bronchiectasis on benralizumab effectiveness. As a common routine practice, the diagnosis of bronchiectasis at baseline in our study was based on the demonstration of airway dilatation on a Chest CT scan. However, bronchial dilatation in severe asthma may be a consequence of poor control of the underlying chronic inflammation resulting in the accumulation of mucus plugs and hyperinflation rather than being a real remodeling of the bronchial walls and caliber [39]. OCS-dependence is a clear index of lack of asthma control, while reduced FEF_25–75%_, is a sign of inflammation of the small airways. In our experience, benralizumab has been shown to give excellent results both in eliminating the need for OCS and in improving the reduction of airflow related to small airways. These results may probably explain why patients who presented these conditions at baseline obtained the greatest benefits from treatment. Additionally, McIntosh et al. [40] recently observed that a significant decrease of the mucus score under benralizumab treatment was accompanied by a parallel improvement in lung function, ventilation and asthma control. As a further hypothesis, therefore, the benralizumab-mediated dissolution of mucus plugs may have contributed to the achievement of long-term favorable outcomes in our patients. Furthermore, these evidences could suggest that bronchietasis in SEA patients may be prevented with early treatment directed at the underlying disease with benralizumab. Moreover, in our center we have already observed that targeting IL-5 in severe eosinophilic asthma with bronchiectasis may be a good therapeutic strategy [41]. Further studies evaluating the persistence or not of bronchial dilation on chest CT before and after treatment with benralizumab would be interesting. The positive association between complete remission and comorbid osteoporosis may also be linked to that with OCS-dependence. Indeed, it is known that SEA patients who are forced to continuously or frequently use OCS to maintain control are burdened by important long-term side effects including osteoporosis [3]. Another positive predictive factor for long-term complete remission appeared to be a predicted FEV_1_% _pre-bd_ ≥ 80%. Generally, it has been observed that patients with less severe lung impairment at initiation had a greater chance of achieving remission with all biologics [42,43].

Therefore, complete remission could be more difficult to achieve in case of a more serious disease with declined lung function and in which remodeling phenomena have already occurred. On this basis, Perez-de-Llano et al. [43] suggested that biologic treatment should not be delayed if remission is the goal. That said, the inclusion of lung function values in the definition of clinical remission in severe asthma is currently under debate due to the multiple asthma and non-asthma related factors (i.e., lung aging) associated with lung function decline [44]. Anyway, in our study baseline clinical characteristics indicated a severely impaired SEA patient population with a predicted FEV_1_ of 66.52 ± 15.94% and benralizumab significantly increased FEV_1_ over time, with predicted levels reaching normal values up to 48 months.

The main limitations of this study lie in its retrospective nature and the relatively small sample size which don’t allow the results obtained to be generalized. For example, the use of paired *t*-tests to compare countable variables between the baseline (T_0_) and the different study points (T_12_, T_24_, T_36_, T_48_) assume normal distribution of differences, which is often questionable in small samples. Furthermore, logistic regression overestimates odds ratios in studies with small to moderate samples size. However, these limitations arise from the design of a real-life experience in a single accredited severe asthma centre. Despite the retrospective data collection doesn’t allow us to exclude possible biases and confounding factors, it allowed us to range within the broader 4-years follow-up period to identify predictors of long-term complete remission. Another limitation of our study may be the lack of an untreated control group to exclude a “placebo effect”. However, using a placebo in real-life doesn’t appear ethical because of the current availably of numerous approved and effective treatments for patients with severe asthma. Together with the long follow-up period, the main strength of our study was the real-world nature of the reported data, as it allowed us to evaluate benefits of benralizumab and its predictors of efficacy in patients with conditions far from the ideals ones established by randomized clinical trials. Consequently, this led us to obtain efficacy information closer to routine clinical practice, although our results need to be confirmed by further studies.

## 5. Conclusions

In conclusion, results of our real-life experience suggest that treatment with benralizumab may allow the achievement and the maintenance of clinical remission in a high percentage of SEA over a follow-up period of 48 months. A long-term 4-criteria outcome of complete clinical remission was more likely to be achieved by SEA patients with comorbid nasal polyposis, probably due to the mutual influence and sharing of the same pathogenetic mechanisms between these two inflammatory airway diseases. Other factors positively associated to complete remission at T_48_ resulted comorbid osteoporosis and bronchiectasis, as well as OCS-dependency, a predicted pre-bronchodilation FEV_1_% ≥ 80% and a predicted FEF_25–75%_ < 65% at baseline. As a take-home-message for clinicians after our 4-years experience with benralizumab, we would like to highlight that clinical remission seems to be a rather dynamic clinical condition, thus underlining the importance of long-term monitoring of asthma patients undergoing biological treatment. A running reassessment of the patient’s inflammatory profile is always suggested to avoid and exclude the attenuation of the effectiveness of a biologic therapy in the long-term. Other studies in real-life and on larger series are certainly needed to confirm our results and beneficial to provide practical information in conditions other than the ideal ones of clinical trials on the achievement of clinical remission in asthma with biologics. At the same time, asthma guidelines should in the future include a precise definition of clinical remission that clinicians and researchers can pursue as a treatment goal.

## Figures and Tables

**Table 1:** Baseline characteristics of the 23 SEA patients included in the study.

Characteristics	Results (*n* = 23)
Age, years (mean ± SD)	58.30 ± 10.26
Females, *n* (%)	16 (69.6%)
Males, *n* (%)	7 (30.4%)
Smokers (ex or current), *n* (%)	5 (21.7%)
BMI, kg/m^2^ (mean ± SD)	27.25 ± 5.00
Asthma onset, years (mean ± SD)	34.26 ± 12.95
Childhood onset, *n* (%)	6 (26.1%)
Adult onset, *n* (%)	17 (73.9%)
Atopy, *n* (%)	17 (73.9%)
Nasal polyposis, *n* (%)	14 (60.9%)
Gastroesophageal reflux, *n* (%)	10 (43.5%)
ASA sensitivity, *n* (%)	3 (13.0%)
Bronchiectasis, *n* (%)	8 (34.8%)
Anxiety and/or depression, *n* (%)	3 (13.0%)
Osteoporosis, *n* (%)	12 (52.2%)
OSAS, *n* (%)	2 (8.7%)
Atopic dermatitis, *n* (%)	2 (8.7%)
Exacerbation patients, *n* (%)	21 (91.3%)
Exacerbation/year, *n* (mean ± SD)	2.61 ± 1.33
OCS-dependent patients, *n* (%)	13 (56.5%)
OCS dosage, mg (mean ± SD)	18.48 ± 12.85
ACT score (mean ± SD)	14.57 ± 2.45
FEV_1_%_pre-bd_, (mean ± SD)	66.52 ± 15.94
FEV_1_ L_pre-bd_ (mean ± SD)	1.95 ± 0.62
FEF_25–75%_ (mean ± SD)	36.83 ± 11.50
Eosinophils, cell/mcL (mean ± SD)	670.87 ± 237.26
Eosinophils > 300 cell/mcL, *n* (%)	22 (95.6%)
FeNO_50_, ppb (mean ± SD)	48.43 ± 14.79
FeNO_50_ > 50 ppb, *n* (%)	9 (39.1%)
Total IgE, kU/L (mean ± SD)	235.18 ± 219.65

Abbreviations: BMI = body mass index; ASA sensitivity = sensitivity to aspirin; OSAS = obstructive sleep apnea syndrome; ACT = asthma control test; FEV_1_ = forced expiratory volume in 1 s; FEF_25–75%_ = forced expiratory flow between 25% and 75% of vital capacity; FeNO_50_ = fractional exhaled nitric oxide at a flow rate of 50 mL/s; IgE = immunoglobulin E.

**Table 2:** Effect of benralizumab on individual outcomes.

Clinical Outcomes	T_0_	T_12_	T_24_	T_36_	T_48_
Exacerbations; mean ± SD (range)	2.61 ± 1.33 (0–6)	0.30 ± 0.45 (0–2)	0.22 ± 0.34 (0–2)	0.09 ± 0.16 (0–1)	0.13 ± 0.23 (0–1)
	*p*-value (T_0_ vs. T_12_) < 0.0001 *				
	*p*-value (T_0_ vs. T_24_) < 0.0001 *				
	*p*-value (T_0_ vs. T_36_) < 0.0001 *				
	*p*-value (T_0_ vs. T_48_) < 0.0001 *				
Exacerbation patients (*n*, %)	21 (91.3%)	4 (17.4%)	3 (23.1%)	1 (4.3%)	3 (23.1%)
	*p*-value (T_0_ vs. T_12_) < 0.0001 *				
	*p*-value (T_0_ vs. T_24_) < 0.0001 *				
	*p*-value (T_0_ vs. T_36_) < 0.0001 *				
	*p*-value (T_0_ vs. T_48_) < 0.0001 *				
Prednisone dose, mg; mean ± SD	18.48 ± 12.85	3.48 ± 5.75	2.17 ± 3.97	1.09 ± 2.08	1.61 ± 2.94
	*p*-value (T_0_ vs. T_12_) 0.0002 *				
	*p*-value (T_0_ vs. T_24_) < 0.0001 *				
	*p*-value (T_0_ vs. T_36_) < 0.0001 *				
	*p*-value (T_0_ vs. T_48_) < 0.0001 *				
Patients requiring OCS (*n*, %)	13 (56.5%)	4 (17.4%)	3 (13.0%)	1 (4.3%)	2 (8.7%)
	*p*-value (T_0_ vs. T_12_) 0.01 *				
	*p*-value (T_0_ vs. T_24_) 0.0045 *				
	*p*-value (T_0_ vs. T_36_) 0.0002 *				
	*p*-value (T_0_ vs. T_48_) 0.001 *				
FEV_1pre-bd_ %; mean ± SD	66.52 ± 15.94	81.30 ± 15.44	81.35 ± 17.81	85.39 ± 14.93	87.14 ± 15.32
	*p*-value (T_0_ vs. T_12_) 0.02 *				
	*p*-value (T_0_ vs. T_24_) 0.02 *				
	*p*-value (T_0_ vs. T_36_) 0.002 *				
	*p*-value (T_0_ vs. T_48_) 0.0008 *				
FEV_1_ gain, mL; mean ± SD	-	296 ± 257	617 ± 273	891 ± 270	1145 ± 258
	*p*-value (T_0_ vs. T_12_) 0.07 *				
	*p*-value (T_0_ vs. T_24_) 0.03 *				
	*p*-value (T_0_ vs. T_36_) 0.002 *				
	*p*-value (T_0_ vs. T_48_) < 0.0001 *				
FEF_25–75%_; mean ± SD	36.83 ± 11.50	53.55 ± 24.28	59.95 ± 26.04	63.05 ± 19.78	66.21 ± 20.79
	*p*-value (T_0_ vs. T_12_) 0.02 *				
	*p*-value (T_0_ vs. T_24_) 0.006 *				
	*p*-value (T_0_ vs. T_36_) < 0.0001 *				
	*p*-value (T_0_ vs. T_48_) < 0.0001 *				
ACT score; mean ± SD	14.57 ± 2.45	22.30 ± 2.00	23.35 ± 1.58	24.39 ± 0.79	24.52 ± 0.75
	*p*-value (T_0_ vs. T_12_) < 0.0001 *				
	*p*-value (T_0_ vs. T_24_) < 0.0001 *				
	*p*-value (T_0_ vs. T_36_) < 0.0001 *				
	*p*-value (T_0_ vs. T_48_) < 0.0001 *				

Abbreviations: T_0_ = baseline; T_12_ = 12 months; T_24_ = 24 months; T_36_ = 36 months; T_48_ = 48 months; OCS = oral corticosteroids, FEV_1_ = forced expiratory volume in 1 s; FEF_25–75%_ = forced expiratory flow between 25% and 75% of vital capacity; ACT = asthma control test score. Statistically significant differences vs. baseline are highlighted with *.

**Table 3:** Effect of benralizumab on inflammation markers.

Markers	T_0_	T_12_	T_24_	T_36_	T_48_
Eosinophils, cell/mcL; mean ± SD	670.87 ± 237.26	52.57 ± 89.12	3.91 ± 6.81	0.87 ± 1.66	0.87 ± 1.66
	*p*-value (T_0_ vs. T_12_) < 0.0001 *				
	*p*-value (T_0_ vs. T_24_) < 0.0001 *				
	*p*-value (T_0_ vs. T_36_) < 0.0001 *				
	*p*-value (T_0_ vs. T_48_) < 0.0001 *				
FeNO_50_, ppb; mean ± SD	48.43 ± 14.79	28.83 ± 11.93	21.38 ± 7.83	16.75 ± 4.76	14.61 ± 4.76
	*p*-value (T_0_ vs. T_12_) 0.002 *				
	*p*-value (T_0_ vs. T_24_) < 0.0001 *				
	*p*-value (T_0_ vs. T_36_) < 0.0001 *				
	*p*-value (T_0_ vs. T_48_) < 0.0001 *				
Total IgE, kU/L; mean ± SD	235.18 ± 219.65	114.19 ± 109.20	65.17 ± 43.84	52.88 ± 36.97	37.78 ± 22.87
	*p*-value (T_0_ vs. T_12_) 0.21				
	*p*-value (T_0_ vs. T_24_) 0.06				
	*p*-value (T_0_ vs. T_36_) 0.04 *				
	*p*-value (T_0_ vs. T_48_) 0.02 *				

Abbreviations: T_0_ = baseline; T_12_ = 12 months; T_24_ = 24 months; T_36_ = 36 months; T_48_ = 48 months; FeNO_50_ = fractional exhaled nitric oxide at a flow rate of 50 mL/s; IgE = immunoglobulin E. Statistically significant differences vs. baseline are highlighted with *.

**Table 4:** Baseline predictors for clinical remission (complete and partial) vs. no remission at T_48_.

Characteristics at T_0_	Complete Remission at T_48_		OR	95% CI		*p*-Value
	Yes (*n* = 15)	No (*n* = 6)				
Females	8 (53.3%)	6 (100.0%)	0.09	0.01	1.82	0.06
Males	7 (46.7%)	0 (0.0%)	11.47	0.55	239.80	0.06
Smokers (past or current)	2 (13.3%)	2 (33.3%)	0.31	0.03	2.94	0.54
BMI > 30 kg/m^2^	4 (26.7%)	2 (33.3%)	0.36	0.04	3.52	0.56
Adult-onset asthma	11 (73.3%)	4 (66.7%)	1.37	0.18	10.66	1.00
Atopy	11 (73.3%)	4 (66.7%)	1.37	0.18	10.66	1.00
Gastroesophageal reflux	6 (40.0%)	4 (66.7%)	0.33	0.04	2.43	0.36
Nasal polyposis	12 (80.0%)	1 (16.7%)	20.00	1.65	241.90	0.01 *
Anxiety and/or depression	2 (13.3%)	1 (16.7%)	0.77	0.06	10.50	1.00
ASA sensitivity	2 (12.3%)	1 (16.7%)	0.77	0.06	10.50	1.00
Osteoporosis	11 (73.3%)	1 (16.7%)	13.75	1.21	156.70	0.04 *
OSAS	2 (13.3%)	0 (0.0%)	2.41	0.10	57.78	1.00
Bronchiectasis	8 (53.3%)	0 (0.0%)	14.73	0.70	308.10	0.04 *
Atopic dermatitis	0 (0.0%)	1 (16.7%)	0.12	0.01	3.26	0.28
≥2 exacerbations/year	11 (73.3%)	5 (83.3%)	0.55	0.05	6.27	1.00
OCS dependency	11 (73.3%)	1 (16.7%)	13.75	1.21	156.70	0.04 *
FEV_1_%_pre-bd_ ≥ 80%	14 (93.3%)	3 (50.0%)	14.00	1.06	185.60	0.05 *
FEF_25–75%_ < 65%	15 (100.0%)	3 (50.0%)	31.00	1.28	747.60	0.01 *
Eosinophils > 300 cell/mcL	15 (100.0%)	5 (83.3%)	8.45	0.30	240.00	0.28
FeNO_50_ > 50 ppb	7 (46.7%)	2 (33.3%)	1.75	0.24	12.65	0.66

Abbreviations: BMI = body mass index; ASA sensitivity = sensitivity to aspirin; OSAS = obstructive sleep apnea syndrome; FEV_1_ = forced expiratory volume in 1 s; FEF_25–75%_ = forced expiratory flow between 25% and 75% of vital capacity; FeNO_50_ = fractional exhaled nitric oxide at a flow rate of 50 mL/s. Statistically significant differences vs. baseline are highlighted with *.

## Data Availability

The data presented in this study are available on request from the corresponding author.
